# A qualitative thematic comparative analysis of independent and AI-supported clinical reflections among medical interns

**DOI:** 10.3389/fmed.2026.1942949

**Published:** 2026-09-15

**Authors:** Selçuk Akturan, Sinan Paslı, Salman Yousuf Guraya

**Affiliations:** 1 Department of Medical Education, Karadeniz Technical University Faculty of Medicine, Trabzon, Türkiye; 2 Department of Emergancy Medicine, Karadeniz Technical University Faculty of Medicine, Trabzon, Türkiye; 3 Collage of Medicine, Gulf Medical University, Ajman, United Arab Emirates

**Keywords:** artificial intelligence, chatbot, professional identity formation, reflective practice, reflective writing

## Abstract

**Background:**

Reflection is fundamental to medical education, supporting clinical reasoning, professional identity formation, and lifelong learning. Although generative artificial intelligence (AI) chatbots have emerged as tools to facilitate reflection, little is known about how AI-supported reflection differs from independent reflective writing. This study compared the characteristics and educational contributions of independent reflective writing and AI-supported reflection among medical interns.

**Methods:**

A comparative qualitative quasi-experimental study was conducted with medical interns during an emergency medicine rotation. The control group was instructed to perform reflective writing independently, while the intervention group was tasked to use AI-supported reflections using a purpose-built chatbot to complete reflective writing. Data were analyzed using qualitative content analysis and compared in terms of themes, reflective depth, clinical reasoning, emotional awareness, and professional learning.

**Results:**

Twenty-one interns completed independent reflective writing, while eighteen engaged in AI-supported reflection using a chatbot. Although both approaches supported reflective learning, they facilitated distinct dimensions of the reflective process. Independent reflective writing was characterized by greater autonomous meaning-making, critical reflection, ethical reasoning, and systems-level thinking. In contrast, AI-supported reflection was characterized by greater emotional articulation, structured reflective dialogue, and future learning planning, but showed more limited exploration of contextual, ethical, and systems-level issues. Participants’ comments embedded within the chatbot interactions suggested that some found the interaction supportive, while repetitive prompting and limited critical exploration emerged as limitations.

**Conclusions:**

Independent reflective writing and AI-supported reflections represent complementary rather than interchangeable educational approaches. While independent reflective writing fosters deeper critical reflection, AI primarily functions as a reflective scaffold that supports emotional engagement and structures reflective learning. Integrating AI-assisted reflections with independent writing and faculty-guided debriefing may optimize reflective learning in medical education.

## 1 Introduction

Reflection is widely recognized as a fundamental pedagogical process in medical education, promoting deeper learning, enhancing clinical reasoning, and supporting the development of professional identity. Schön's (1983) concepts of reflection-in-action and reflection-on-action provide a theoretical foundation for integrating experiential learning with clinical practice (1). Foundational and more recent research has consistently highlighted the role of reflection in fostering self-regulated learning, ethical sensitivity, empathy, and lifelong learning skills (2–5).

The internship year represents a critical transition in medical education, during which clinical responsibility increases substantially, and professional roles become more clearly defined. During this period, medical interns encounter high workloads, time pressure, emotionally demanding situations, and increasing responsibility for patient care (6, 7). In this context, reflection is particularly important for helping interns make sense of clinical experiences, improve clinical decision-making, process emotional experiences, and support their professional development (8).

Reflective writing is one of the most widely used approaches for facilitating reflection in clinical education. By encouraging learners to describe, analyze, and critically evaluate their clinical experiences, reflective writing may facilitate deeper learning by enabling them to connect experiences with existing knowledge, examine their actions and underlying assumptions, identify areas for improvement, and develop greater self-awareness (9). However, reflective writing does not always achieve the desired depth because of time constraints, limited feedback, and declining learner motivation (10–12).

Recent advances in artificial intelligence (AI) and natural language processing have created new opportunities to support reflective learning through digital technologies. In particular, AI-powered chatbots can guide learners through structured reflective questioning and provide individualized support throughout the reflective process (13). Such technologies have the potential to make reflective practice more accessible and sustainable within the demanding environment of clinical education.

Despite these promising developments, the educational quality and depth of AI-supported reflection remain insufficiently understood. Concerns have been raised that algorithm-driven guidance may inadvertently limit learners’ critical thinking, autonomous meaning-making, and internal reflective dialogue, potentially resulting in more superficial reflection (14, 15). Moreover, empirical studies directly comparing independent reflective writing with AI-supported reflection remain scarce, particularly in undergraduate medical education.

This study aimed to explore how independent reflective writing and chatbot-based AI-supported reflection differ in the quality, depth, and nature of reflection among medical interns. It also aimed to examine how the components of reflection were manifested across the two approaches and to identify interns’ perceptions and experiences of AI-supported reflection as embedded within their reflective narratives and chatbot dialogues.

## 2 Materials and methods

### 2.1 Study design

This study employed a comparative quasi-experimental qualitative design to compare two pedagogical approaches to reflection among medical interns: independent reflective writing and AI-supported reflection using a chatbot. Random allocation was not undertaken because the reflective activities were embedded within the existing emergency medicine rotation and implemented at the rotation-group level rather than individually. Individual randomization would have required different reflective activities to be delivered simultaneously to interns within the same clinical rotation, creating practical difficulties in implementation and increasing the potential for cross-group interaction and contamination. Therefore, the study was conducted within the existing rotation structure to preserve the natural educational context.

### 2.2 Study setting

The study was conducted from 15 April 2026 to 15 June 2026 at the Department of Emergency Medicine at Karadeniz Technical University Hospital. The emergency department provides a particularly relevant context for reflective learning because learners frequently encounter diagnostic uncertainty, rapid clinical decision-making, emotionally challenging experiences, ethical dilemmas, and the need to function effectively under pressure (16, 17). During this rotation, medical interns actively participated in patient assessment, initial management, interprofessional communication, and patient safety activities, providing clinically meaningful experiences for subsequent reflection.

### 2.3 Participants and sampling

Medical interns undertaking their emergency medicine rotation during the study period were eligible to participate. A purposive convenience sampling technique was employed. Participation was voluntary, and written informed consent was obtained from all participants. Participants were allocated according to their pre-existing emergency medicine rotation groups rather than through individual randomization. Interns in the first rotation completed independent reflective writing, whereas those in the second rotation completed AI-supported reflection using the chatbot. These rotation groups had been established as part of the routine undergraduate clinical schedule independently of the study.

A total of 39 medical interns participated in the study. Of these, 21 completed independent reflective writing, whereas 18 participated in AI-supported reflection using the chatbot.

### 2.4 Study procedure

This study was conducted in five steps:

#### 2.4.1 Step 1. Development of the AI-supported reflection interface

A custom web-based reflection application was developed specifically for this study using Python. The application incorporated a purpose-built generative AI chatbot powered by the GPT-5 model through the OpenAI API to provide real-time, structured AI-supported reflective dialogue. It was designed to facilitate structured reflection while providing a simple and intuitive user interface. Participants accessed the platform through a web browser, entered their study identification number, and initiated a reflection session. The user interface consisted of a conversational chat window organized around the sequential stages of reflection and a free-text input area through which participants entered their reflections. Upon clicking the “Start Session” button, participants were presented with a pop-up warning instructing them not to enter any personally identifying information about themselves, patients, or any other individuals into the application (e.g., names, medical record numbers, or other identifying information). A representative screenshot of the application interface is provided in Appendix 1.

Each participant response was transmitted via the OpenAI API to the GPT-5 model together with a predefined system prompt that specified the chatbot's educational role, communication style, conversation rules, and reflective framework; no patient records, medical record numbers, photographs, or other clinical documents were transmitted through the application. The system prompt was a standardized set of instructions provided to the AI model at the beginning of each interaction to define its role, establish the rules and boundaries of the dialogue, and specify how it should guide participants through the reflective process. It instructed the model to function as a reflective facilitator by asking one open-ended question at a time, encouraging independent meaning-making, and concluding the dialogue with a summary of the learner's reflections. The reflective framework embedded within the system prompt was developed based on Gibbs’ Reflective Cycle, incorporating its six sequential stages: description, feelings, evaluation, analysis, conclusion, and action plan (18). The complete system prompt and reflective framework are presented in Supplementary Material 1.

Based on participants’ responses, the AI generated context-specific follow-up questions while maintaining consistency with the predefined reflective framework. All interactions were presented in a conversational chat format within an application developed exclusively to standardize the AI-supported reflection process. To ensure data privacy, the complete reflection transcripts were de-identified and exported in docx format for qualitative analysis. These files were stored on a secure, password-protected institutional computer accessible exclusively to the research team, and will be retained for five years following publication in accordance with institutional research data management policies. Furthermore, OpenAI API calls were configured with data storage disabled (*store* *=* *false*), ensuring that participant responses were not retained through the API for model storage.

#### 2.4.2 Step 2. Orientation and preparation

Before data collection, all participants received information regarding the study objectives, procedures, voluntary participation, confidentiality, and data protection. Importantly, all participants had previously completed the same mandatory one-week module on reflection and reflective writing during the fifth year of the undergraduate medical curriculum. This module, required for all fifth-year medical students, included the theoretical foundations of reflection, structured approaches to reflection, and practical reflective writing activities. Thus, both groups entered the study with the same formal curricular preparation in reflection and reflective writing.

Prior to the reflection activities in the present study, brief refresher sessions were provided to both groups based on this previously acquired knowledge. For the independent reflective writing group, the 3-hour refresher session focused primarily on recalling the principles and process of reflective writing. The refresher was structured around Gibbs’ Reflective Cycle, consistent with the reflective framework underlying the AI-supported tool. During the session, participants were provided with a Gibbs-based reflective writing guide that had previously been developed and used as part of the mandatory fifth-year reflection module. Copies of this guide were provided to participants, and they were informed that they could refer to it while completing their reflective writing. Immediately after the refresher session, participants were given one hour to independently write a reflection on a meaningful clinical experience, after which the handwritten reflections were collected by the researchers.

For the AI-supported reflection group, the 2-hour refresher session focused on recalling the fundamental concepts of reflection and how to engage in the reflective process. This refresher was similarly based on the same Gibbs-informed reflective principles. These sessions were intended to refresh previously learned concepts rather than provide new or additional pedagogical training. Participants in the AI-supported reflection group also received a 1-hour technical orientation on how to access and use the chatbot.

#### 2.4.3 Step 3. Group allocation

This study employed a non-randomized, quasi-experimental cohort design. During the study period, medical interns completed their mandatory emergency medicine rotation in successive clinical rotation groups that had been scheduled by the faculty before the study commenced. Students were invited to participate voluntarily in the research; however, participation in the study did not influence group allocation. Instead, each existing rotation cohort was assigned to a single reflection modality according to the predetermined study schedule. One cohort completed independent reflective writing (control), whereas the subsequent cohort completed AI-supported reflection using the chatbot (intervention). Participants were therefore allocated according to their pre-existing clinical rotation cohorts rather than individual preference or self-selection, within the established curricular structure of the mandatory emergency medicine rotation.

#### 2.4.4 Step 4. Reflection activities

During the emergency medicine rotation, participants were asked to reflect on a meaningful clinical experience, such as a challenging patient encounter, diagnostic uncertainty, an ethical dilemma, communication difficulties within the healthcare team, or a patient safety incident.

Participants in the control group completed reflective writings independently using the reflective framework described above. Participants in the intervention group completed the same reflective task using the purpose-built AI chatbot, which was structured around the same reflective framework. Rather than providing reflective content or suggested responses, the chatbot facilitated the reflective process by generating adaptive, structured follow-up questions based on participants’ responses, supporting further exploration of their experiences.

#### 2.4.5 Step 5. Data collection

For the independent reflective writing group, reflective writings were handwritten on paper and securely retained in their original form. These written narratives constituted the data used for qualitative analysis. For the AI-supported reflection group, the chatbot generated an individual report containing the complete reflective dialogue for each participant, which was exported and stored in docx format and used for qualitative analysis. All data were de-identified before analysis to ensure participant anonymity. Each participant completed one reflection, resulting in one reflective narrative or one chatbot-generated reflection report per participant.

### 2.5 Data analysis

Data were analyzed using qualitative content analysis, following the approaches described by Graneheim and Lundman (2004) and Elo and Kyngäs (2008) (19, 20).

For the AI-supported group, the unit of analysis consisted exclusively of participants’ written responses within the chatbot dialogues. Chatbot-generated questions, prompts, summaries, and other AI-generated text were not coded or included in the assessment of reflective depth, clinical reasoning, emotional awareness, or professional learning. The complete dialogues were retained only to preserve the conversational context in which participants’ responses were generated.

No separate survey, questionnaire, interview, or focus group was administered at any stage of the study. Accordingly, all findings relating to participants’ perceptions and feedback regarding the chatbot were derived exclusively from evaluative remarks that participants spontaneously embedded within their own responses during the chatbot dialogues, and were neither solicited by the chatbot nor prompted by the researchers. These remarks were identified inductively during coding, whenever a participant's response referred to the reflection tool itself, for example, to the helpfulness of the questions, the repetitiveness of the prompting, or the experience of interacting with the chatbot, rather than to the clinical experience being reflected upon. Because such remarks arose spontaneously and were not elicited systematically, they are reported as interaction-embedded perceptions rather than as a formal evaluation of usability, satisfaction, or acceptability, and no claim is made that they are representative of the group as a whole. The analysis consisted of the following steps:

1. Repeated reading of the transcripts
2. Identification of descriptions
3. Open coding
4. Generation of subthemes and overarching themes
5. Comparative analysis between the two reflection approaches

Coding was conducted independently by two researchers. Discrepancies were resolved through discussion until consensus was achieved.

Reflective depth was evaluated using the framework proposed by Wald et al. (2012), which conceptualizes reflection as a continuum ranging from descriptive to analytical and critical reflection (9). Each participant's reflection was examined individually for evidence of descriptive, analytical, and critical reflective processes. Descriptive reflection was identified when participants primarily recounted clinical events; analytical reflection when they interpreted their experiences, explored underlying causes, or considered alternative perspectives; and critical reflection when they questioned assumptions, integrated emotions and values, considered contextual or systems-level influences, or identified implications for future professional practice.

In addition to reflective depth, each reflection was coded for dimensions of clinical reasoning, emotional awareness, and professional learning. Following this individual-level analysis, coded patterns were compared across the two groups to identify differences in their recurrence and prominence. These comparisons were qualitative and pattern-based rather than based on statistical frequency testing. Accordingly, comparative terms used in the Results, such as “deeper,” “greater,” and “less frequently observed,” refer to recurring qualitative patterns identified across individual reflections within each group rather than quantitative effect estimates.

### 2.6 Ethics declaration

This study was approved by the Scientific Research Ethics Committee of Karadeniz Technical University Faculty of Medicine (Protocol No. 2026/67; Approval Date: 08 April 2026). Written informed consent was obtained from all participants prior to participation. The manuscript contains no identifiable personal information; therefore, additional consent for publication was not required.

## 3 Results

A total of 21 independent reflective writings and 18 AI-supported reflection dialogues conducted through the chatbot were analyzed thematically. Although several dimensions of reflection were evident across both approaches, differences emerged in the nature, scope, and depth of reflective thinking (Figure 1).

**Figure 1:**
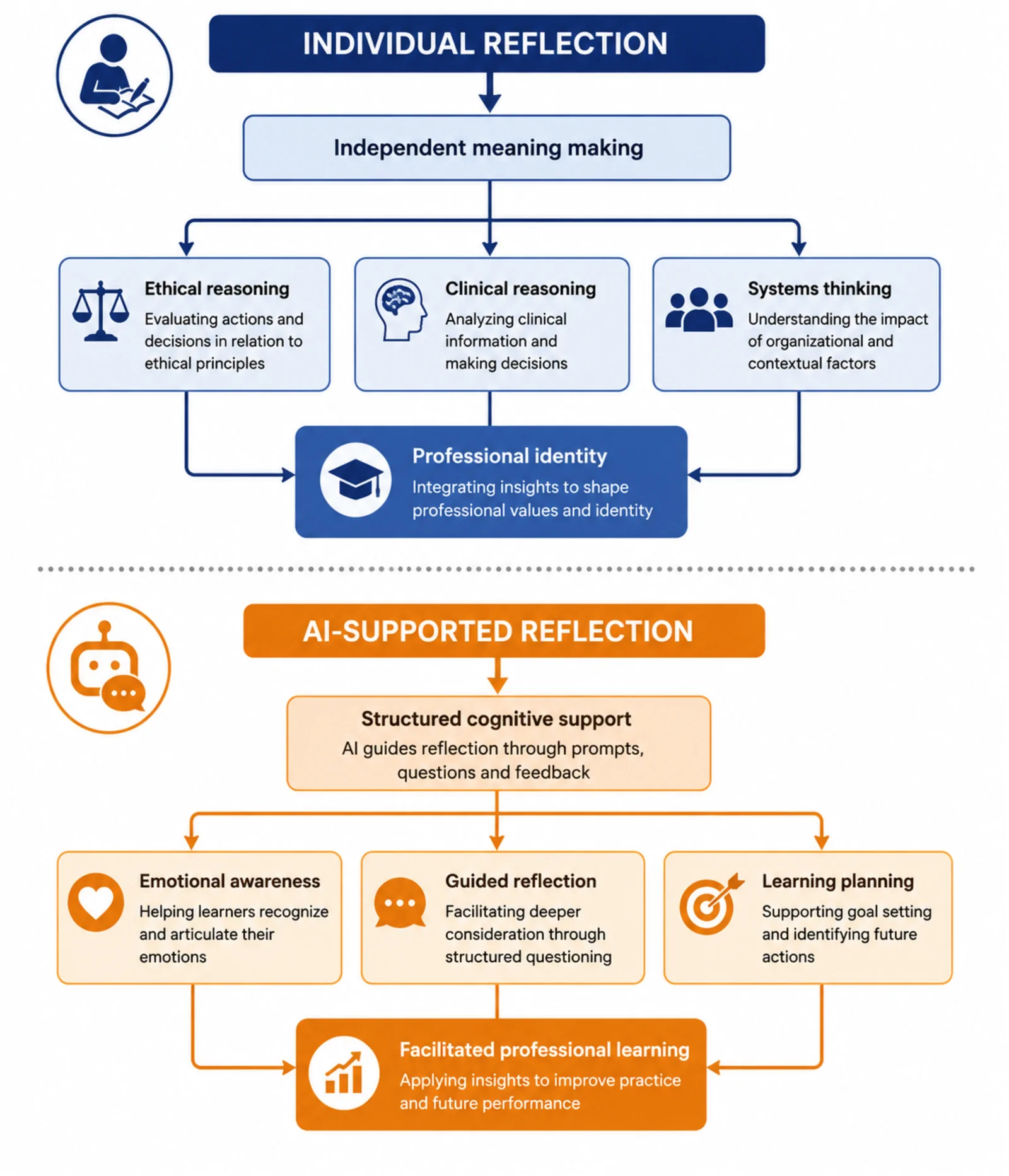
Conceptual pathways of independent reflective writing and AI-supported reflection in professional learning.

In line with the comparative aim of the study, the themes identify what participants in each group chose to reflect on, whereas the reflective dimensions examined thereafter show how they reflected on it. The themes are therefore reported first as the content-level basis of the comparison.

Four overarching themes emerged from the independent reflective writings: (1) reconstructing patient-centered care, (2) the strength and fragility of clinical communication, (3) development of professional identity through self-awareness, and (4) the influence of the healthcare system on professional behavior. These themes reflected not only the clinical issues addressed by participants but also how they interpreted and critically examined their experiences. Independent reflections were characterized particularly by consideration of patient welfare, ethical dilemmas, personal responsibility, team dynamics, and system-related challenges.

Six themes emerged from the AI-supported reflections: (1) complexity of the clinical environment and workload, (2) emotional awareness, (3) patient communication, (4) team communication, (5) professional identity development, and (6) future learning plans. These themes reflected a comparatively greater emphasis on emotional processing, organization of clinical experiences, and identification of future learning needs. However, system-level issues, ethical reasoning, and multidimensional contextual analysis were less prominent than in the independent reflections.

Overall, while both approaches incorporated patient communication, professional development, and interpretation of clinical experiences, they differed in the dimensions of reflective thinking that were most prominent. These differences were further examined in terms of reflective depth, clinical reasoning, emotional awareness, and professional learning.

These thematic differences map directly onto the dimensions examined below. In the independent reflective writings, the emphasis on ethical dilemmas, team dynamics, and system-related constraints corresponds to the greater reflective depth and systems-level clinical reasoning, and the focus on professional identity to the more self-directed professional learning; in the AI-supported reflections, the prominence of emotional awareness and workload corresponds to the richer emotional articulation, while the absence of an ethics- or system-oriented theme mirrors the more limited contextual reasoning. What follows therefore does not present a separate set of findings but examines how these thematic differences were expressed within the reflective process itself.

### 3.1 Reflective depth

Independent reflective writing generally demonstrated deeper levels of reflection, with students independently questioning their thought processes, considering alternative actions, and critically evaluating the ethical dimensions of clinical situations. Rather than merely describing what had happened, participants explored why events occurred, reflected on their own roles, and considered how they would act differently in similar situations in the future.

In contrast, although the chatbot facilitated reflections, the reflective process was largely guided by standardized prompts and repeated questions such as:

- "What did you learn from this experience?”
- "What would you do differently next time?”
- "How did this experience affect you?”

Although this process effectively initiated reflections, it appeared to limit the natural progression of deeper inquiry over time. Consequently, independent reflections were characterized by more spontaneous and authentic reflective processes, whereas chatbot-supported reflections followed a more structured and template-driven pattern.

### 3.2 Clinical reasoning

Independent reflections extended beyond clinical decision-making to include consideration of alternative treatment options, team-based decisions, organizational processes, and ethical consequences.

For example, one participant stated:

“I believe the problems that occurred in this process resulted from poor communication between departments.” (RW-5)

Similarly, another participant noted:

“Under no circumstances should a patient suffer because of someone’s stubbornness.” (RW-7)

These excerpts illustrate students’ ability to analyze clinical events from a systems perspective.

In contrast, chatbot-supported reflections focused primarily on patient communication, empathy, and individual behaviors. Comparisons of alternative clinical decisions, consideration of different team perspectives, and systems-level reasoning were less frequently observed.

### 3.3 Emotional awareness

The greatest difference between the two approaches was observed in emotional awareness, favoring chatbot-supported reflection. The chatbot encouraged students to identify, explain, and elaborate on their emotional responses and to reflect on the personal impact of clinical experiences. Its supportive and non-judgmental responses appeared to facilitate emotional disclosure. However, these responses represented programmed conversational strategies rather than genuine empathic understanding.

“It is completely understandable that you felt this way in such a situation.”

appeared to facilitate emotional disclosure.

Although emotionally rich reflections were also witnessed in independent writings, these expressions emerged more spontaneously and were initiated by the students themselves rather than being prompted externally.

### 3.4 Professional learning

Both approaches supported learners in generating insights for future professional practice. However, in independent reflective writing these learning outcomes emerged primarily through learners’ own self-evaluation and critical reflection. In contrast, professional learning in chatbot-supported reflection generally developed following the chatbot's guiding questions.

Accordingly, independent reflective writing fostered more self-directed professional learning, whereas the chatbot primarily functioned as a cognitive facilitator that supported learners in organizing and extending their reflective thinking.

### 3.5 Interaction-embedded perceptions of the chatbot

Analysis of the AI-supported reflection dialogues revealed both positive and critical perceptions of the chatbot-supported reflection process that were embedded within participants’ interactions. These perceptions were not obtained through a separate survey, questionnaire, or interview; they were drawn exclusively from evaluative remarks that participants introduced spontaneously while responding to the chatbot. They are therefore reported as qualitative observations arising from the dialogue data rather than as systematically elicited evaluative feedback.

Three positive themes emerged:

- Facilitating the initiation of reflection
- Stimulating thinking
- Perceived supportiveness of the interaction

Participants’ statements within the chatbot dialogues indicated that the structured interaction helped them organize their experiences and consider alternative perspectives.

However, we identified four major limitations: excessive guidance, repetitive questioning, insufficient contextual and systems-level inquiry, and failure to complete the reflective cycle through detailed planning.

Overall, interaction-embedded comments suggested that the chatbot provided cognitive support by facilitating the initiation and organization of reflection and encouraging emotional expression. Nevertheless, it did not consistently enable the level of deep reflection required for learners to construct their own independent meanings and interpretations.

## 4 Discussion

This study demonstrates that independent reflective writing and AI-supported reflection facilitate different dimensions of reflective learning rather than representing competing educational approaches. Independent reflective writing primarily promoted autonomous meaning-making and critical reflection, whereas AI-supported reflection facilitated emotional engagement and structured reflective dialogue. These findings showcase unique and new insights into the pedagogical role of generative AI in reflective practice. The following sections describe themes generated from our study with an analysis of our findings with the published literature.

### 4.1 Independent reflective writing promotes autonomous meaning-making and professional identity formation

Our findings indicate that independent reflective writing promotes a qualitatively different learning process by encouraging learners to construct meaning without external guidance. Students spontaneously questioned their clinical decisions, examined ethical dilemmas, considered alternative management strategies, reflected on organizational influences, and critically evaluated their own professional roles. These findings are consistent with Kegan's constructive-developmental theory, which conceptualizes learning as an active process of meaning-making, and Baxter Magolda's concept of self-authorship, whereby learners progressively shift from relying on external authority to developing internally constructed professional judgment (21, 22).

The independently written reflections also reflected characteristics of transformative learning described by Mezirow (1997), as learners moved beyond describing events to questioning assumptions, examining ethical issues, and reconsidering future actions (23). Consistent with previous research, reflective writing appeared to strengthen reflective capacity by supporting independent professional judgement and professional identity formation (3, 9, 24). Unlike previous AI studies that primarily evaluated feedback quality or AI-generated reflective texts (25, 26), our findings demonstrate that independent reflective writing engages a distinct learning mechanism centered on autonomous meaning-making.

### 4.2 AI supports reflection through cognitive scaffolding but has limited capacity to promote critical reflection

The present findings suggest that AI-supported reflection functions primarily as a form of reflective scaffolding. Through structured questioning, summarizing learners’ responses, validating emotions, and encouraging future planning, the chatbot supported learners in organizing their reflections and sustaining reflective dialogue. This pattern closely resembles instructional scaffolding described by Wood et al. (1976) and aligns with Vygotsky's concept of guided learning within the Zone of Proximal Development, which describes the space between what a learner can accomplish independently and what they can achieve with appropriate guidance or support (27, 28).

Consistent with emerging evidence, AI provided structured, accessible, and supportive guidance throughout the reflective process (25). However, unlike previous work in which ChatGPT primarily provided feedback on reflections that students had already written independently, the present study examined AI as an active conversational partner that scaffolded reflection itself. This distinction is noteworthy because recent systematic evidence indicates that, despite the growing use of AI-driven simulation to enhance communication, clinical reasoning, empathy, and decision-making, reflection and reflective practice have not yet been examined as educational outcomes (29). Moreover, although generative AI appears to enhance learner engagement, it may be less effective in fostering higher-order critical thinking (30). Our findings extend this emerging literature by suggesting that AI's principal educational contribution lies in scaffolding reflective dialogue—supporting emotional expression, organizing experiences, and prompting future-oriented learning—rather than replacing reflective practice itself. At the same time, the deepest processes of meaning-making, critical reflection, and professional identity formation appeared to remain predominantly learner-driven.

The present findings also help explain why this distinction matters. Although AI effectively guided learners through describing experiences and organizing their reflections, its scaffolding remained largely procedural rather than transformative. Unless learners themselves introduced ethical tensions, organizational influences, conflicting perspectives, or broader system-level factors, AI rarely prompted deeper interrogation of these dimensions. Consequently, reflections frequently remained at descriptive or affective levels instead of progressing toward critical reflection characterized by questioning underlying assumptions, examining contextual influences, and reconstructing meaning. These findings reinforce the view that AI currently functions best as a facilitator of reflective engagement rather than as a catalyst for the complex cognitive and interpretive processes required for critical reflection. However, this limitation should not necessarily be regarded as an inherent limitation of AI-supported reflection. As generative AI models and pedagogically designed prompting strategies evolve, future systems may be better able to recognize opportunities for deeper inquiry and actively prompt learners to examine ethical tensions, underlying assumptions, contextual influences, and systems-level factors. More sophisticated adaptive reflective frameworks may therefore enable AI to support dimensions of critical reflection that were less evident in the present study. Until such capabilities are empirically demonstrated, however, independent or human-facilitated reflection remains important for supporting deeper epistemic, ethical, and identity-forming dimensions of reflective practice.

### 4.3 Emotional articulation alone does not ensure reflective depth

One of the most distinctive findings was AI's ability to facilitate emotional articulation. Compared with independent reflective writing, learners described their emotions more explicitly, explored their causes, and reflected more openly on the personal impact of clinical experiences. Interaction-embedded comments suggested that some participants found the AI-facilitated reflection supportive.

Nevertheless, richer emotional expression did not necessarily translate into deeper reflective inquiry. Discussions seldom progressed toward examining underlying assumptions, ethical tensions, or broader organizational and system-level influences. As Boud et al. (1985) argued, emotions provide an important starting point for reflection but are insufficient on their own to achieve transformative learning (31). Similarly, reflection in health professions education requires contextual inquiry and systems thinking in addition to emotional awareness (2, 32, 33). Our findings therefore suggest that AI is particularly effective in facilitating emotional engagement but may require complementary educational strategies or human facilitation to promote deeper critical reflection.

### 4.4 Educational implications

Taken together, these findings suggest that independent reflective writing and AI-supported reflection should not be regarded as competing educational approaches but rather as complementary educational strategies that cultivate different dimensions of reflective learning. Independent reflective writing appears to promote autonomous meaning-making, critical reflection, and professional identity formation, whereas AI-supported reflection facilitates reflective engagement by helping learners organize experiences, articulate emotions, and identify initial learning goals. These complementary roles are consistent with recent perspectives advocating for the integration of generative AI into health professions education as a pedagogical partner that augments, rather than replaces, human teaching, learner agency, and faculty mentorship (29, 34). Integrating AI-supported dialogue with independent reflective writing and faculty-facilitated debriefing may therefore offer a more comprehensive model for reflective learning, combining the scalability and structured cognitive scaffolding afforded by AI with the uniquely human capacities for critical dialogue, contextual interpretation, ethical reasoning, and professional identity formation fostered through independent reflection and educator guidance. Such an integrated approach may enable learners to benefit from the strengths of both modalities while preserving the depth and transformative potential that characterize reflective practice in health professions education.

### 4.5 Limitations

This study has several limitations. The relatively small sample of medical interns from a single institution and specialty may limit the transferability of the findings. Allocation by pre-existing rotation cohorts rather than individual randomization may have introduced cohort effects or unmeasured baseline differences. Additionally, baseline participant characteristics and prior experience with generative AI or chatbots were not systematically collected, limiting our ability to assess group comparability on these potentially relevant factors. Although both groups had the same prior curricular training and Gibbs-informed framework, immediate preparation differed: the independent writing group received a three-hour reflective writing refresher, whereas the AI-supported group received a two-hour reflection refresher plus one hour of technical orientation, potentially influencing reflective engagement and depth. Participants’ awareness of the study and the novelty of the AI-supported approach may also have influenced engagement. The findings are also specific to the AI model, system prompt, and reflective framework used and should therefore be interpreted within this context. The use of an externally hosted AI model also presents data-governance limitations, as reflective narratives may contain clinically sensitive contextual information despite the exclusion of direct identifiers. Future studies could consider locally hosted or institutionally controlled AI models to provide greater control over data processing, storage, and access. Finally, participants’ perceptions of the chatbot were not assessed using a dedicated instrument. Because these perceptions were captured only through remarks that arose spontaneously within the dialogues, they represent the views of those participants who chose to comment rather than of the group as a whole, and they should not be interpreted as a measure of usability, satisfaction, or acceptability. Future studies combining dialogue analysis with a validated evaluation instrument or post-session interviews would allow these perceptions to be examined more systematically.

## 5 Conclusion

This study provides important insights into how independent reflective writing and AI-supported reflection among medical interns may function as complementary rather than interchangeable approaches to reflective learning in medical education. Independent reflective writing promotes autonomous meaning-making, critical reflection, systems thinking, and professional identity formation, whereas AI-supported reflection primarily functions as a form of reflective scaffolding by facilitating emotional articulation and structuring reflective dialogue. Although AI effectively supports reflective engagement, it does not adequately promote contextual, ethical, and systems-level inquiry required for deeper critical reflection. These findings propose that the educational value of generative AI lies in complementing and not replacing independent reflections and faculty facilitation. By comparing the reflective processes generated through independent reflective writing and AI-supported reflections, our study showcases the current understanding of how different reflective modalities shape and navigate learning processes. Future research is essential to explore hybrid models that integrate AI-supported dialogues with independent reflective writing to maximize both emotional engagement and critical reflection.

## Data Availability

The raw data supporting the conclusions of this article will be made available by the authors, without undue reservation.

## Appendix 1

The application interface.

## References

[1] Schön DA. The Reflective Practitioner: How Professionals Think in Action. New York: Basic Books (1983).

[2] Mann K Gordon J MacLeod A. Reflection and reflective practice in health professions education: a systematic review. Adv Health Sci Educ. (2009) 14(4):595–621. 10.1007/s10459-007-9090-2 18034364

[3] Sandars J. The use of reflection in medical education: AMEE guide No. 44. Med Teach. (2009) 31(8):685–95. 10.1080/01421590903050374 19811204

[4] Koh S Townsend J. Teaching self-regulated learning through reflective writing: experiences of first-year medical students with the master adaptive learner model. Cureus. (2024) 16(10):e72664. 10.7759/cureus.72664 39618585 PMC11606618

[5] Hashim MJ Khan MAB Kieu A Alkaabi FMK Al Rubaei FKA Alfazari MMK. Using reflective writing to enhance empathy among medical students. Can Med Educ J. (2024) 15(2):91–2. 10.36834/cmej.77172 38827898 PMC11139784

[6] Jia X Du J Xu T Yin X Zhang Q Bao X. Investigating the status and influencing factors of professional identity formation among Chinese medical interns: a mixed methods study. BMC Med Educ. (2025) 25(1):676. 10.1186/s12909-025-07199-3 40340896 PMC12063465

[7] Zhou J Jin L Hu Y. Mechanism of career resilience formation during the role transition of medical interns: a grounded theory study. Front Med (Lausanne). (2025) 12:1671520. 10.3389/fmed.2025.1671520 41333782 PMC12665529

[8] Forrest RM Iden TC Edwards C Kimberly C Foster KW Lee B. Making reflective practice by interns visible through digital storytelling. J Gen Intern Med. (2026) 41(7):1855–66. 10.1007/s11606-026-10217-9 41741861 PMC13176442

[9] Wald HS Borkan JM Taylor JS Anthony D Reis SP. Fostering and evaluating reflective capacity in medical education: developing the REFLECT rubric for assessing reflective writing. Acad Med. (2012) 87(1):41–50. 10.1097/ACM.0b013e31823b55fa 22104060

[10] Sandars J Cleary TJ. Self-regulation theory: applications to medical education. AMEE guide No. 58. Med Teach. (2011) 33(11):875–86. 10.3109/0142159X.2011.595434 22022899

[11] Stranges TN MacNutt MJ. Reflective writing assignments in the era of GenAI: student behavior and attitudes suggest utility, not futility. Adv Physiol Educ. (2025) 49(2):582–92. 10.1152/advan.00241.2024 40247747

[12] Kawakami C Nomura O Takahashi M Takaha R Ito T Ushikoshi H. Rapidly rising preference among new-entry medical students for using generative artificial intelligence in reflective reports. JMA Journal. (2025) 8(2):465–9. 10.31662/jmaj.2024-0390 40415990 PMC12095134

[13] Chan KS Zary N. Applications and challenges of implementing artificial intelligence in medical education: integrative review. JMIR Med Educ. (2019) 5(1):e13930. 10.2196/13930 31199295 PMC6598417

[14] Bearman M Molloy E Ajjawi R Keating J. Is there a plan B? Clinical educators supporting medical students in uncertainty. Med Educ. (2020) 54(6):516–26. 10.1111/medu.14116

[15] Topol E. Deep Medicine: How Artificial Intelligence can Make Healthcare Human Again. London: Hachette UK (2019).

[16] Bernard AW Gorgas D Greenberger S Jacques A Khandelwal S. The use of reflection in emergency medicine education. Acad Emerg Med. (2012) 19(8):978–82. 10.1111/j.1553-2712.2012.01407.x 22818356

[17] Pandit K Chu DL Russell R Wright M Titone L Diaz T. The utility of an online discussion board for reflective writing in an emergency medicine rotation. AEM Educ Train. (2023) 7(4):e10899. 10.1002/aet2.10899 37529174 PMC10387829

[18] Gibbs G. Learning by Doing: A Guide to Teaching and Learning Methods. Oxford: Further Education Unit, Oxford Polytechnic (1988).

[19] Graneheim UH Lundman B. Qualitative content analysis in nursing research: concepts, procedures and measures to achieve trustworthiness. Nurse Educ Today. (2004) 24(2):105–12. 10.1016/j.nedt.2003.10.001 14769454

[20] Elo S Kyngäs H. The qualitative content analysis process. J Adv Nurs. (2008) 62(1):107–15. 10.1111/j.1365-2648.2007.04569.x 18352969

[21] Kegan R. In Over our Heads: The Mental Demands of Modern Life. Cambridge, MA: Harvard University Press (1994).

[22] Magolda MB King PM. Learning Partnerships: Theories and Models of Practice to Educate for Self-Authorship. Sterling, VA: Stylus Publishing (2004).

[23] Mezirow J. Transformative learning: theory to practice. New Dir Adult Contin Educ. (1997) 1997:5–12. 10.1002/ace.7401

[24] Avargil S Saleh A Kerem NC. Exploring aspects of medical students’ professional identity through their reflective expressions. BMC Med Educ. (2025) 25(1):1111. 10.1186/s12909-025-07611-y 40713614 PMC12291310

[25] Haider N Morjaria L Sheth U Al-Jabouri N Sibbald M. MD student perceptions of ChatGPT for reflective writing feedback in undergraduate medical education. Int Med Educ. (2025) 4(3):27. 10.3390/ime4030027

[26] Wraith C Carnegy A Brown C Baptista A Sam AH. Can educators distinguish between medical student and generative AI-authored reflections? Med Educ. (2025) 59(11):1214–21. 10.1111/medu.15750 40600244 PMC12513547

[27] Wood D Bruner JS Ross G. The role of tutoring in problem solving. J Child Psychol Psychiatry. (1976) 17(2):89–100. 10.1111/j.1469-7610.1976.tb00381.x 932126

[28] Vasileva O Balyasnikova N. (Re)introducing Vygotsky’s thought: from historical overview to contemporary psychology. Front Psychol. (2019) 10:1515. 10.3389/fpsyg.2019.01515 31447717 PMC6692430

[29] Loubbairi S Moussaoui YE Lahlou L Chakri I Nassik H. The impact of artificial intelligence-driven simulation on the development of non-technical skills in medical education: a systematic review. J Educ Eval Health Prof. (2025) 22:37. 10.3352/jeehp.2025.22.37 41566844

[30] Pham TD Karunaratne N Exintaris B Liu D Lay T Yuriev E. The impact of generative AI on health professional education: a systematic review in the context of student learning. Med Educ. (2025) 59(12):1280–9. 10.1111/medu.15746 40533396 PMC12686775

[31] Boud D Keogh R Walker D. Reflection: Turning Experience into Learning. London: Routledge (1985).

[32] Plack MM Driscoll M Marquez M Cuppernull L Greenberg L. A method for assessing reflective journal writing. J Allied Health. (2005) 34(4):199–208. 16529182

[33] Weller-Newton JM Drummond-Young M. Reflective practice in health professions education. In: Nestel D Reedy G McKenna L Gough S, editors. Clinical Education for the Health Professions. Singapore: Springer (2023:441–62). 10.1007/978-981-15-3344-0_32

[34] Hersh W. Generative artificial intelligence: implications for biomedical and health professions education. Annu Rev Biomed Data Sci. (2025) 8(1):355–80. 10.1146/annurev-biodatasci-103123-094756 40203232

